# Cold atmospheric plasma inactivates Aspergillus flavus and Fusarium keratoplasticum biofilms and conidia in vitro

**DOI:** 10.1099/jmm.0.001858

**Published:** 2024-07-10

**Authors:** Darby Roberts, Jonathan Thomas, Jacklyn Salmon, Marc A. Cubeta, Katharina Stapelmann, Brian C. Gilger

**Affiliations:** 1 Department of Clinical Sciences, College of Veterinary Medicine, NC State University, Raleigh, NC, USA; 2 Department of Nuclear Engineering, College of Engineering, NC State University, Raleigh, NC, USA; 3 Department of Entomology and Plant Pathology, College of Agriculture and Life Science, NC State University, Center for Integrated Fungal Research, Raleigh, NC, USA

**Keywords:** antifungal, *Aspergillus*, cold atmospheric plasma, *Fusarium*, fungal disease

## Abstract

**Introduction.***Aspergillus flavus* and *Fusarium keratoplasticum* are common causative pathogens of fungal keratitis (FK), a severe corneal disease associated with significant morbidity and vision loss. Escalating incidence of antifungal resistance to available antifungal drugs poses a major challenge to FK treatment. Cold atmospheric plasma (CAP) is a pioneering nonpharmacologic antimicrobial intervention that has demonstrated potential as a broad-spectrum antifungal treatment.

**Gap statement.** Previous research highlights biofilm-associated resistance as a critical barrier to effective FK treatment. Although CAP has shown promise against various fungal infections, its efficacy against biofilm and conidial forms of FK pathogens remains inadequately explored.

**Aim.** This study aims to investigate the antifungal efficacy of CAP against clinical fungal keratitis isolates of *A. flavus* and *F. keratoplasticum in vitro*.

**Methodology.** Power parameters (22–27 kV_pp_, 300–400 Hz and 20–80 mA) of a dielectric barrier discharge CAP device were optimized for inactivation of *A. flavus* biofilms. Optimal applied voltage and total current were applied to *F. keratoplasticum* biofilms and conidial suspensions of *A. flavus* and *F. keratoplasticum*. The antifungal effect of CAP treatment was investigated by evaluating fungal viability through means of metabolic activity, c.f.u. enumeration (c.f.u. ml^−1^) and biofilm formation.

**Results.** For both fungal species, CAP exhibited strong time-dependent inactivation, achieving greater than 80 % reduction in metabolic activity and c.f.u. ml^−1^ within 300 s or less, and complete inhibition after 600 s of treatment.

**Conclusion.** Our findings indicate that CAP is a promising broad-spectrum antifungal intervention. CAP treatment effectively reduces fungal viability in both biofilm and conidial suspension cultures of *A. flavus* and *F. keratoplasticum*, suggesting its potential as an alternative treatment strategy for fungal keratitis.

## Introduction

Fungal keratitis (FK) is an infectious corneal disease with the potential to cause blindness in upwards of 2 million people and numerous animals each year [1,6]. Caused by exposure to widespread environmental fungal pathogens, commonly species of *Aspergillus* and *Fusarium*, FK is characterized by severe pain, limited response to medical treatment and a high risk of vision loss in the affected eye [7,8]. Once considered a rare opportunistic infection, FK now accounts for 6–53 % of all infectious ulcerations in humans and 20–57 % of infectious ulcerations in horses, the most affected veterinary species [9,12]. This escalation in FK incidence, attributed to factors such as environmental change and a growing population of immunosuppressed individuals, is paralleled by concerning development of resistance among the causative organisms to the limited arsenal of available antifungal agents [3,12,15]. Antifungal resistance is especially common in species of *Fusarium* [15,18].

Recently, there has been increased interest in the role of biofilm formation as a resistance mechanism in pathogenic filamentous fungi [19]. Biofilms are adherent multicellular microbial communities embedded within an extracellular matrix that confers enhanced antimicrobial tolerance compared to planktonic conidia or hyphae [20,22]. Biofilm formation has been described as a contributing factor to the pathogenicity and resistance of clinical FK isolates *in vitro* [23,25]. In a study investigating *in vitro* antifungal susceptibility of *A. fumigatus*, Mowat *et al*. [23] demonstrated that biofilm formation resulted in enhanced resistance to antifungals of three distinct modes of action, including voriconazole, a frontline and commonly used therapeutic agent. Similarly, *in vitro* biofilm formation and associated antifungal resistance have been demonstrated in clinical FK isolates of *F. solani* and *F. oxysporum* [24,25]. These studies concluded that biofilm-associated antifungal resistance is a critical factor driving FK treatment failure, warranting immediate investigation into novel techniques that can effectively inactivate fungal biofilms.

A potential treatment option for mitigating biofilm-associated microbial infections is the use of cold atmospheric plasma (CAP). Generated by applying a low-current high-voltage to a gas, CAP can produce an array of biologically active reactive oxygen and nitrogen species (RONS) without significant thermal output, making it safe to use on host tissues [26]. Early studies investigating CAP as a potential antifungal therapy have demonstrated CAP-induced inactivation of *Aspergillus flavus* spores *in vitro*, enhanced antifungal susceptibility of *Candida* biofilms following CAP treatment *in vitro* and safe and effective application of CAP for reducing corneal fungal burden *in vivo* [27,34]. However, the existing literature on CAP’s antifungal effect in filamentous fungi lacks a thorough examination of biofilms, comprehensive power parameter comparisons and consistency in device design and input, impeding meaningful comparisons of results. Furthermore, to our knowledge, no studies have investigated the effects of CAP on FK-associated isolates of *Fusarium* species.

The purpose of this study is to report on the *in vitro* dose-dependent inactivation of clinical isolates of *A. flavus* and *Fusarium keratoplasticum* treated with CAP exposure. The antifungal effect of CAP treatment was investigated by evaluating fungal viability through means of metabolic activity, c.f.u. enumeration (c.f.u. ml^−1^), and biofilm formation. Initially, the operating parameters of CAP were optimized for the inactivation of *A. flavus* biofilms where the effect of current (20–80 mA), voltage (22, 26 and 27 kV_pp_) and duration (0–300 s) were optimized. Next, optimal power parameters were applied to *F. keratoplasticum* biofilms and conidial (asexual spore) suspensions of *A. flavus* and *F. keratoplasticum*. Ultimately, we report effective CAP inactivation of *A. flavus* and *F. keratoplasticum* biofilms and conidia.

## Methods

### CAP system

**Fig. 1.**
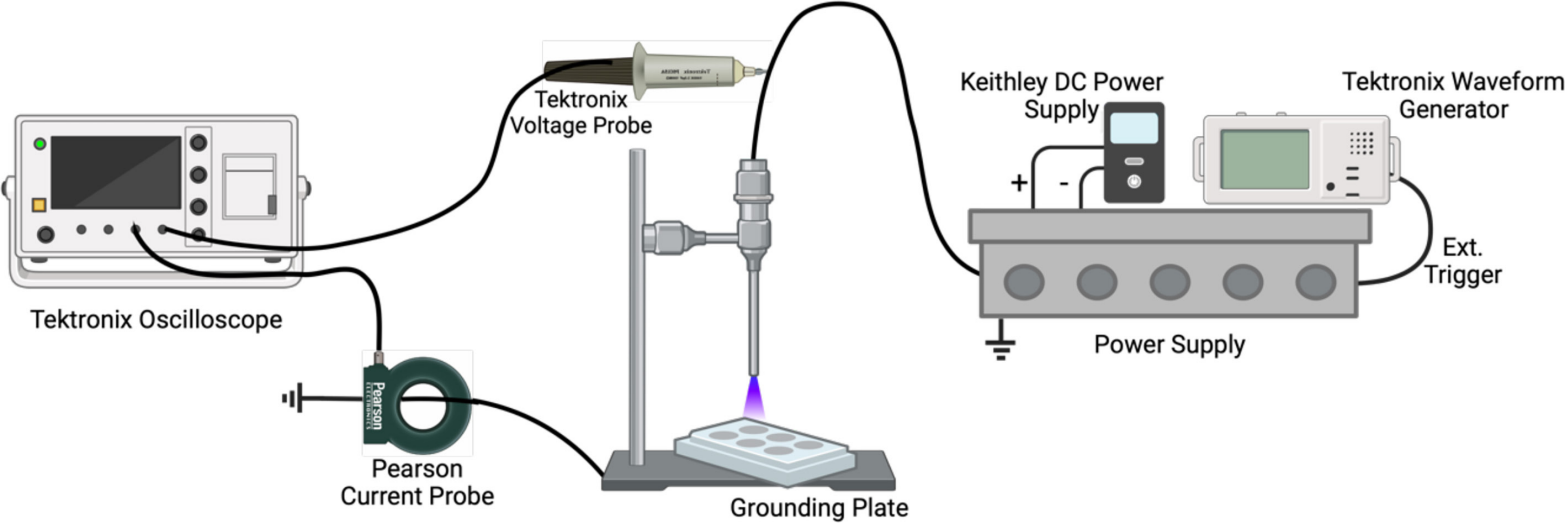
Schematic representation of CAP dielectric barrier discharge (DBD) device and configuration for treating fungal samples. Created with BioRender.com.

The CAP system used in this study was a dielectric barrier discharge device consisting of a cylindrical copper electrode covered by an aluminium oxide dielectric and driven by a self-built microsecond-pulsed power supply [35]. The high-voltage electrode had a diameter of 10 mm. Applied voltage and discharge current measurements were conducted using a high-voltage probe (P6015A, Tektronix, USA), a current monitor (Model 6585, Pearson Electronics, Palo Alto, CA, USA) and a digital oscilloscope (MSO64, Tektronix, Beaverton, ORBeaverton, OR, USA). A representative schematic of the device configuration is depicted in Fig. 1.

**Fig. 2.**
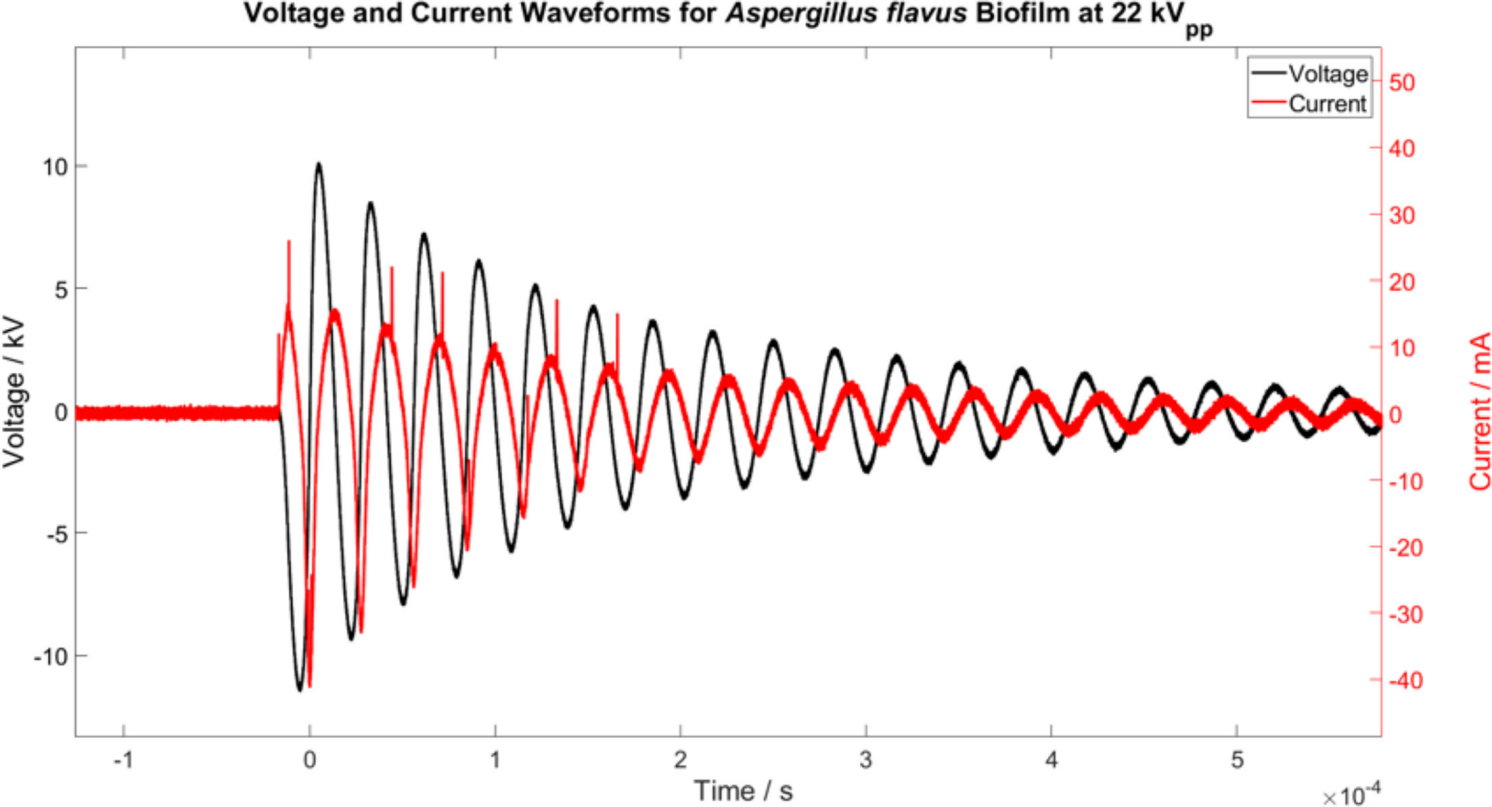
Representative voltage and current waveform. Obtained during CAP treatment of *A. flavus* biofilm at a pulse repetition frequency of 300 Hz. Peak-to-peak voltage is measured at 22 kV_pp_ and total current at 20 mA.

With this CAP device, the pulse repetition frequency can be varied between 100 and 2000 Hz, and the high-voltage amplitude can be set within the range of 5 and 27 kV_pp_. Representative voltage and current waveforms are shown in Fig. 2. Average power per pulse (W) was calculated for select treatments using the following equation: $\underline{P}=\frac{1}{T}\int_{0}^{T}U\left(t\right)∙I\left(t\right)dt$. Dissipated power per pulse (*W*) was calculated by subtracting the measurements obtained while the plasma is ‘on’ by the measurements obtained while the plasma is ‘off’: $\underline{P}$_diss_ (*I*) = $\underline{P}$_on_ (*I*) − $\underline{P}$_off_ (*I*).

### Fungal cultivation

#### Strain and maintenance

Two fungal species, *A. flavus* and *F. keratoplasticum*, were selected for use in this study based on their prevalence in clinical keratitis cases. Clinical isolates were originally collected from equine patients with fungal keratitis presented to NC State College of Veterinary Medicine. After the initial culture sample evaluation and identification, isolates were further cultured for purification; at such time, single conidial-derived stocks were prepared and stored frozen at −80 °C in 2 ml cryogenic vials until the time of use [36]. Multi-locus sequence typing (MLST) was performed to confirm species identification and identify evolutionary lineage and/or species haplotype [36]. The *A. flavus* isolate used for this study was identified as MLST designation AF9 lineage subgroup IC, while the *F. keratoplasticum* isolate used was identified as MLST designation FK1 haplotype 2 u [36].

#### Asexual spore preparation

Isolates of *A. flavus* and *F. keratoplasticum* used for this study were recovered from −80 °C on Potato Dextrose Agar (PDA, Difco, Franklin Lakes, NJ, USA). Cultures were prepared from frozen conidial stocks by flash-thawing cryovials in a 45 °C water bath until few ice crystals remained, briefly vortexing and spreading 40 µl of the conidial suspension onto PDA. Plates were incubated at 33 °C, 5 % CO_2_ for 3–5 days. Spore suspensions were made by flooding each plate with 3–5 ml potato dextrose broth prepared at a 50 % dilution (PDB50, Difco, Franklin Lakes, NJ, USA). The agar surface was scraped using a sterile rubber spatula, and the resulting suspension was filtered through sterile cheesecloth lining a funnel. The suspension was diluted 1 : 10 with PDB50 and concentration was adjusted to 1×10^5^ conidia per ml with a haemocytometer (Hausser Scientific, Horsham, PA, USA).

#### Biofilm preparation

*A. flavus and F. keratoplasticum* biofilms were prepared in sterile 24-well flat bottom tissue culture plates (Corning Inc., Corning, NY, USA) by adding 150 µl of 1×10^5^ conidia per ml spore suspension and 450 µl of PDB50 per well. Plates were incubated at 33 °C, 5 % CO_2_ for 24 h. After incubation, the medium was aspirated and biofilms were washed three times with 1–2 ml of phosphate-buffered saline (PBS, Thermo Fischer Scientific, Waltham, MA, USA) to remove unattached cells.

### CAP treatment

#### CAP treatment of biofilms

CAP treatments were performed on prewashed *A. flavus* and *F. keratoplasticum* biofilms. An aluminium grounding plate was fitted to the bottom of the culture plate and the dielectric barrier discharge (DBD) electrode was positioned over the centre of sample well, leaving a 1-mm gap distance between the sample surface and electrode tip. *A. flavus* biofilms were treated at 22–27 kV_pp_, 300–500 Hz and 20–80 mA for 0–600 s. *F. keratoplasticum* biofilms were treated at 22 kV_pp_, 20 mA and 300 Hz for 0–600 s. There were three replicates of each treatment.

#### CAP treatment of spores

To perform CAP treatment of *A. flavus* and *F. keratoplasticum* conidia, 150 µl of 1×10^5^ conidia per ml suspension and 150 µl of PDB50 were added to each well in a sterile 24-well flat bottom tissue culture plate (Corning Inc.). An aluminium grounding plate was fitted to the bottom of the culture plate, and the DBD electrode was positioned over the centre of the sample well with a 1.5 mm gap distance between the liquid surface and electrode tip. All treatments were performed at an amplitude voltage of 22 kV, current of 20 mA and repetition frequency of 300 Hz for 0–600 s. There were three replicates of each treatment.

### Fungal cell viability assay

Following CAP treatment of fungal biofilms, 100 µl of PDB50 was added to each plasma-treated well and non-plasma-treated control well. Biofilms were scraped thoroughly with a 1000 µl pipette tip, and the well contents were transferred to a 2 ml round bottom Eppendorf tube and vortexed. The resultant suspensions were subjected to tenfold serially dilutions in PDB50. Plasma-treated and control fungal spore samples were similarly processed by transferring well contents to a round bottom 2 ml Eppendorf tube. All wells were washed with 300 µl PDB50, the wash was transferred to the corresponding Eppendorf tube and resultant suspensions were serially diluted tenfold with PDB50. Aliquots (20 µl) of appropriate dilutions and undiluted samples, as well as uninoculated PDB50 (negative control), were plated onto PDA and incubated at 33 °C, 5 % CO_2_ for 24–48 h. The number of fungal colonies measured as c.f.u. ml^−1^ was recorded.

### Metabolic activity assay

An XTT (2,3-bis-(2-methoxy-4-nitro-5-sulfophenyl)-2H-tetrazolium-5-carboxanilide, disodium salt) assay was performed to quantify the metabolic activity of treated and untreated spore and biofilm samples [27]. XTT (Thermo Fischer Scientific) was prepared at a concentration of 200 µg ml^−1^. Prior to the assay, 2.5 µl of 10 mM menadione (MilliporeSigma, Burlington, MABurlington, MA, USA) was added to the XTT solution to reach a final concentration of 25 µM. Biofilm samples were processed immediately post-treatment, and conidia samples were processed following a 24-h post-treatment incubation period. For the assay, 200 µl of XTT reagent was added per well. Well plates were then incubated at 33 °C, 5 % CO_2_ for 3 h in the dark to form the coloured XTT formazan product. Following incubation, 100 µl supernatant per well was transferred to a clean flat bottom 96-well plate for absorbance reading. The absorbance at 490 and 690 nm wavelength was measured (Varioskan Lux multimode plate reader; Thermo Fischer Scientific). Each absorbance value was corrected by subtracting the 690 nm reference wavelength from the 490 nm absorbance value and then subtracting the mean absorbance (490 nm) of three blank (non-inoculated) wells identically processed.

### Crystal violet assay

To quantify biofilm formation by CAP-treated conidial suspensions, a crystal violet (CV) adhesion assay was performed [27]. Following CAP treatment, treated and untreated control conidia samples were incubated for 24 h at 33 °C, 5 % CO_2_. Subsequently, wells were washed twice with 0.5 ml PBS to remove unadhered cells and 200 µl 0.005 % CV (MilliporeSigmaBurlington, MA) was added to each well. After 15 min, excess CV was removed, and wells were washed four to six times with 0.5 ml PBS. Plates were incubated at 33 °C, 5 % CO_2_ for 3 h to dry, and then 200 µl of 95 % ethanol was added per well. Solubilized CV (100 µl) was transferred per well to a clean flat bottom 96-well plate for absorbance reading at 590 nm (Varioskan Lux multimode plate reader; Thermo Fischer Scientific, Waltham, MA, USA). Each absorbance value was corrected by subtracting the mean absorbance of three blank (non-inoculated) wells identically processed.

### Statistical analysis

ANOVA and Tukey’s post-hoc analysis for multiple mean comparisons were performed to determine significant differences among means at a 95.0 % confidence level (*α*=0.05). Differences were considered significant at *P*≤0.05. All data analyses were performed using GraphPad Prism v10.1.1 for macOS (GraphPad Software, Boston, MA, USA).

## Results

### Optimization of CAP treatment of *A. flavus* biofilms

To investigate the influence of pulse repetition frequency (Hz) on the antifungal effect of CAP, metabolic activity and c.f.u. ml^−1^ of *A. flavus* biofilms was determined following a 300 s duration CAP treatment at 27 kV_pp_, 300 or 400 Hz and 60 mA. The 300 and 400 Hz treatments significantly reduced metabolic activity and c.f.u. ml^−1^ compared to the untreated controls, and there were no significant differences in reduction between the two treatments (Fig. S1a,b). This experiment was repeated, this time with a 180 s CAP treatment at 26 kV_pp_, 400 or 500 Hz and 40 mA. The 400 and 500 Hz treatments resulted in significant reductions in metabolic activity and c.f.u. ml^−1^ with no statistical differences between the two treatments (Fig. S1c,d). Since neither frequency resulted in greater reductions in fungal viability, a pulse repetition frequency of 300 Hz was selected for all subsequent treatments.

**Fig. 3.**
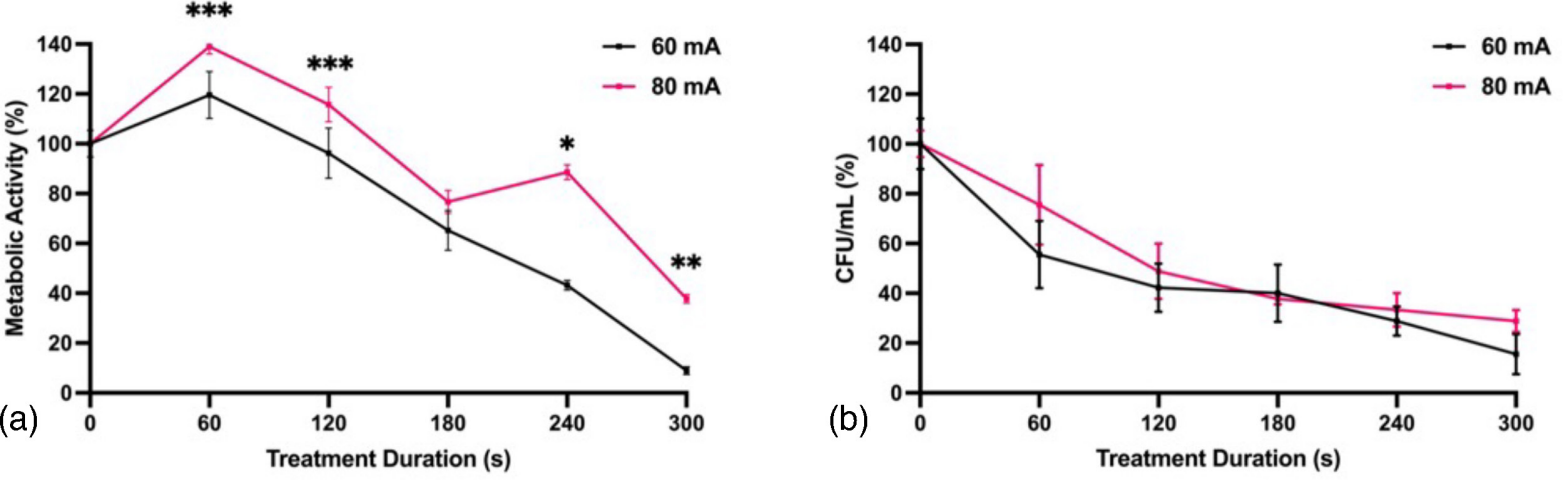
Improved CAP activity against *A. flavus* biofilms at 60 and 80 mA. (**a**) Metabolic activity and (**b**) c.f.u. ml^−1^ following 0, 60, 120, 180, 240 or 300 s CAP treatment at 27 kV_pp_, 300 Hz and 60 or 80 mA. Metabolic activity determined by the XTT reduction with absorbance values normalized to untreated controls. c.f.u. ml^−1^ were normalized to untreated controls. Significant differences between sample groups are indicated; *P<0.0001, **P<0.005, ***P<0.05 (mean±sem; *n*=3).

The effect of electrical current on fungal viability was examined by treating *A. flavus* biofilms with CAP at a peak-to-peak voltage of 25 kV_pp_ and a pulse repetition frequency of 300 Hz for 0, 60, 120, 180, 240 or 300 s at either 60 or 80 mA. Treating at 60 mA resulted in significantly greater reductions in metabolic activity for most treatment durations compared to 80 mA (Fig. 3a). However, there was no significant difference in c.f.u. ml^−1^ between the two treatments (Fig. 3b). These results indicate that pursuing a low operating current may offer the dual benefit of improved fungal inactivation while avoiding the detrimental physiological effects associated with the application of high current to biological tissues [37].

**Fig. 4.**
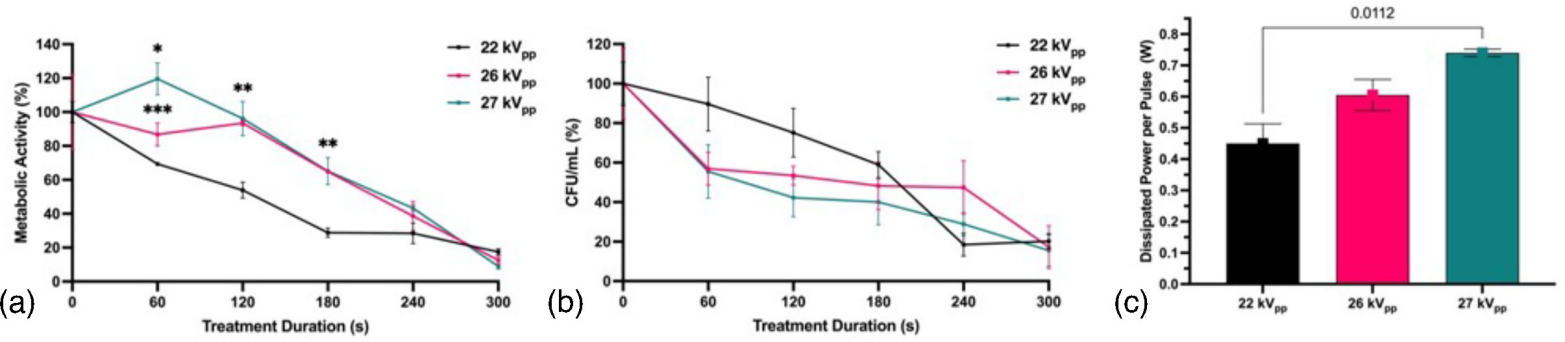
Time- and power-dependent CAP-induced inactivation of *A. flavus* biofilms. (**a**) Metabolic activity, (**b**) c.f.u. ml^−1^ following 0, 60, 120, 180, 240 and 300 s CAP treatment and (**c**) dissipated power per pulse (W) at low (22 kV_pp_, 20 mA), mid (26 kV_pp_, 40 mA) and high (27 kV_pp_, 60 mA) power. A pulse repetition frequency of 300 Hz was applied for all treatments. Metabolic activity determined by XTT reduction with absorbance values normalized to untreated controls. c.f.u. ml^−1^ were normalized to untreated controls. Significant differences from 22 kV_pp_ treatment group are indicated; *P<0.0001, **P<0.005, ***P<0.01 (mean±sem; *n*=3).

The results of time and power (applied voltage) variation demonstrated dose-dependent inactivation of *A. flavus* biofilms (Fig. 4). In general, the reduction in fungal viability improved with lower power inputs and longer treatment durations. Concerning metabolic activity, mid (26 kV_pp_) and high power (27 kV_pp_) treatment resulted in delayed reductions at shorter treatment times of less than 3 min, followed by rapid reduction after treatment for 3 min or more with a maximal reduction in metabolic activity of 91 % after 5 min treatment of 27 kV_pp_ (Fig. 4a). Low-power (22 kV_pp_) treatment revealed a rapid initial reduction in metabolic activity that plateaued after 3 min. With respect to c.f.u. ml^−1^, mid (26 kV_pp_) and high (27 kV_pp_) powers caused rapid initial inactivation of *A. flavus* biofilms followed by a pronounced flattening of the curve over time (Fig. 4b). Low-power (22 kV_pp_) treatment, however, resulted in a more linear response with a maximum 82 % reduction in c.f.u. ml^−1^ after 4 min (Fig. 4b). Treating with low power (22 kV_pp_) resulted in significantly greater reductions in fungal metabolic activity from 1 to 3 min compared to mid or high power. Due to improved reductions in fungal viability at low power, an amplitude voltage of 22 kV_pp_ and corresponding current of 20 mA were selected for all subsequent experiments.

### Effect of CAP on *F. keratoplasticum* biofilm viability

**Fig. 5.**
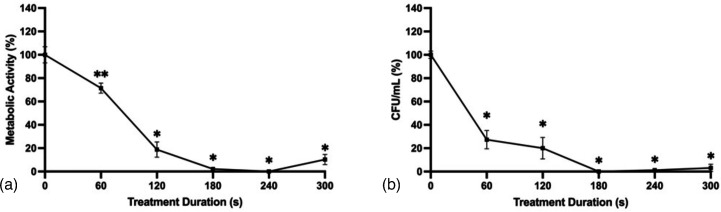
CAP treatment rapidly inactivates *F. keratoplasticum* biofilms *in vitro*. (**a**) Metabolic activity and (**b**) c.f.u. ml^−1^ following 0, 60, 120, 180, 240 or 300 s CAP treatment at 22 kVpp, 300 Hz and 20 mA. Metabolic activity determined by XTT reduction with absorbance values normalized to untreated controls. c.f.u. ml^−1^ were normalized to untreated controls. Significant differences from untreated controls (0 s) are indicated; *P<0.0001, **P<0.01 (mean±sem; *n*=3).

The effect of CAP on *F. keratoplasticum* biofilm viability was investigated at operating parameters of 22 kV_pp_, 300 Hz and 20 mA for 0, 60, 120, 180, 240 or 300 s. Metabolic activity and c.f.u. ml^−1^ were significantly reduced after 60 s of treatment and reached 98 % inhibition after 180 s of treatment (Fig. 5a and b).

### Complete reduction in biofilm viability following CAP treatment

**Fig. 6.**
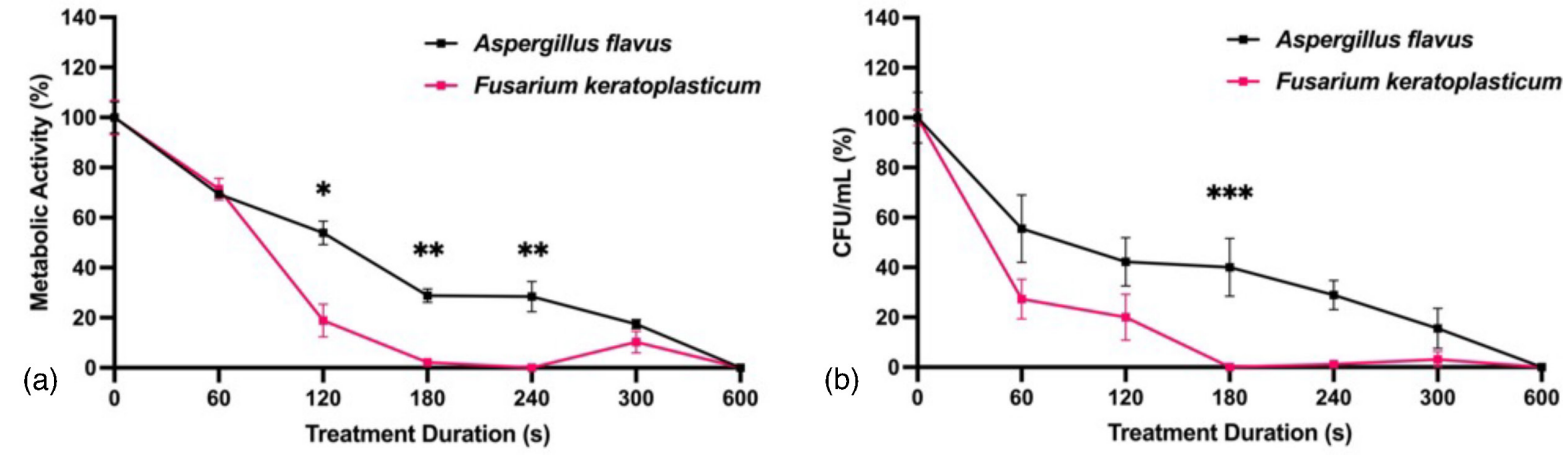
CAP-induced reduction in viability of *A. flavus* and *F. keratoplasticum* biofilms after 600 s treatment. (**a**) Metabolic activity and (**b**) c.f.u. ml^−1^ following 0, 60, 120, 180, 240, 300 or 600 s CAP treatment at 22 kV_pp_, 300 Hz, and 20 mA. Metabolic activity determined by XTT reduction with absorbance values normalized to untreated controls. c.f.u. ml^−1^ were normalized to untreated controls. Significant differences between sample groups shown; *P<0.0001, **P<0.001, ***P<0.01 (mean±sem; *n*=3).

*In vitro* biofilms of *A. flavus* and *F. keratoplasticum* were exposed to CAP at 22 kV_pp_, 300 Hz and 20 mA for 600 s. Complete (100 %) reductions in metabolic activity and c.f.u. ml^−1^ were observed in both fungal species (Fig. 6). CAP-induced inactivation of *F. keratoplasticum* occurred earlier compared to *A. flavus*.

### Antifungal effect of CAP on *A. flavus* spores

**Fig. 7.**
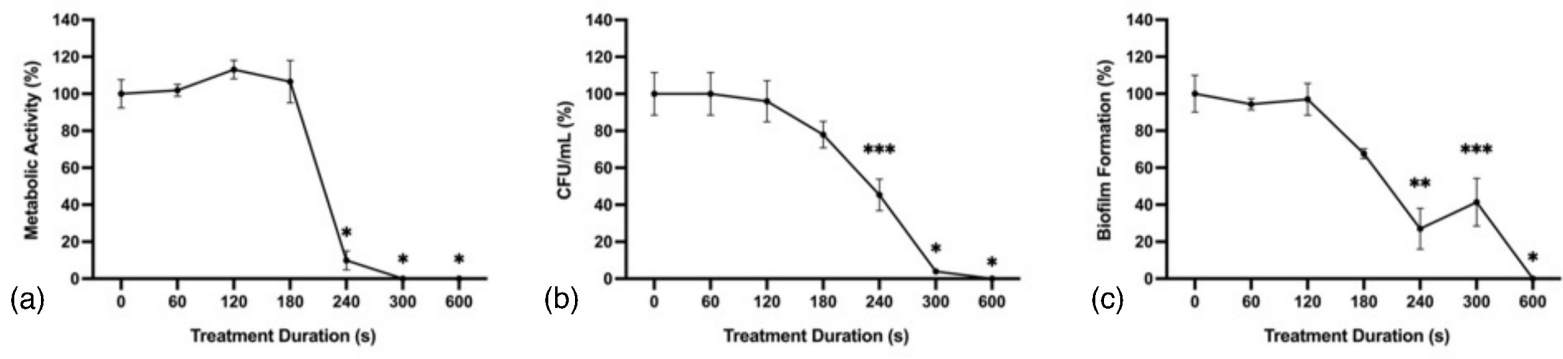
*A. flavus* spore viability following CAP treatment. *A. flavus* conidia treated with CAP for 0, 60, 120, 180, 240, 300 or 600 s at 22 kV_pp_, 300 Hz, and 20 mA. (**a**) Metabolic activity determined 24 h post-treatment by XTT reduction with absorbance values normalized to untreated controls. (**b**) c.f.u. ml^−1^ were normalized to untreated controls. (**c**) Biofilm formation determined by crystal violet uptake with absorbance values normalized to untreated controls. Significant differences from untreated controls (0 s) are indicated; *P<0.0001, **P<0.001, ***P<0.01 (mean±sem; *n*=3).

*A. flavus* conidial suspensions were treated with CAP at 22 kV, 300 Hz and 20 mA for 0, 60, 120, 180, 240, 300 or 600 s. A long delay phase in fungal inactivation was observed, followed by rapid inactivation as treatment times exceeded 180 s (Fig. 7). CAP treatment for 300 s reduced metabolic activity and c.f.u. ml^−1^ by greater than 90 %, while treatment for 600 s resulted in a complete reduction of metabolic activity, c.f.u. ml^−1^, and biofilm formation.

### Antifungal effect of CAP on *F. keratoplasticum* spores

**Fig. 8.**
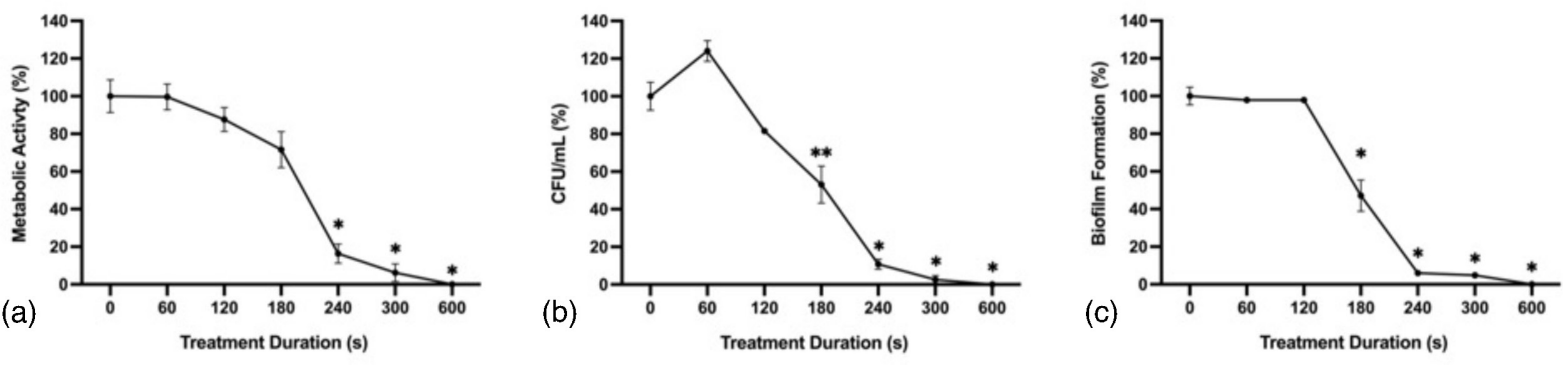
*F. keratoplasticum* spore viability following CAP treatment. *F. keratoplasticum* conidia treated with CAP for 0, 60, 120, 180, 240, 300 and 600 s at 22 kV_pp_, 300 Hz and 20 mA. (**a**) Metabolic activity determined 24 h post-treatment by XTT reduction with absorbance values normalized to untreated controls. (**b**) c.f.u. ml^−1^ were normalized to untreated controls. (**c**) Biofilm formation determined by crystal violet uptake with absorbance values normalized to untreated controls. Significant differences from untreated controls (0 s) are indicated; *P<0.0001, **P<0.001, ***P<0.01 (mean±sem; *n*=3).

*F. keratoplasticum* conidial suspensions were treated with CAP at 22 kV, 300 Hz and 20 mA for 0, 60, 120, 180, 240, 300 or 600 s. Similar to *A. flavus* conidial suspensions, delayed inactivation was observed followed by rapid inactivation after treatment durations of 180 s or more (Fig. 8a–c). CAP treatment for 600 s again resulted in a complete reduction in fungal viability, evidenced by a complete reduction of metabolic activity, c.f.u. ml^−1^ and biofilm formation compared to untreated controls.

### Average dissipated power per fungal specimen

**Fig. 9.**
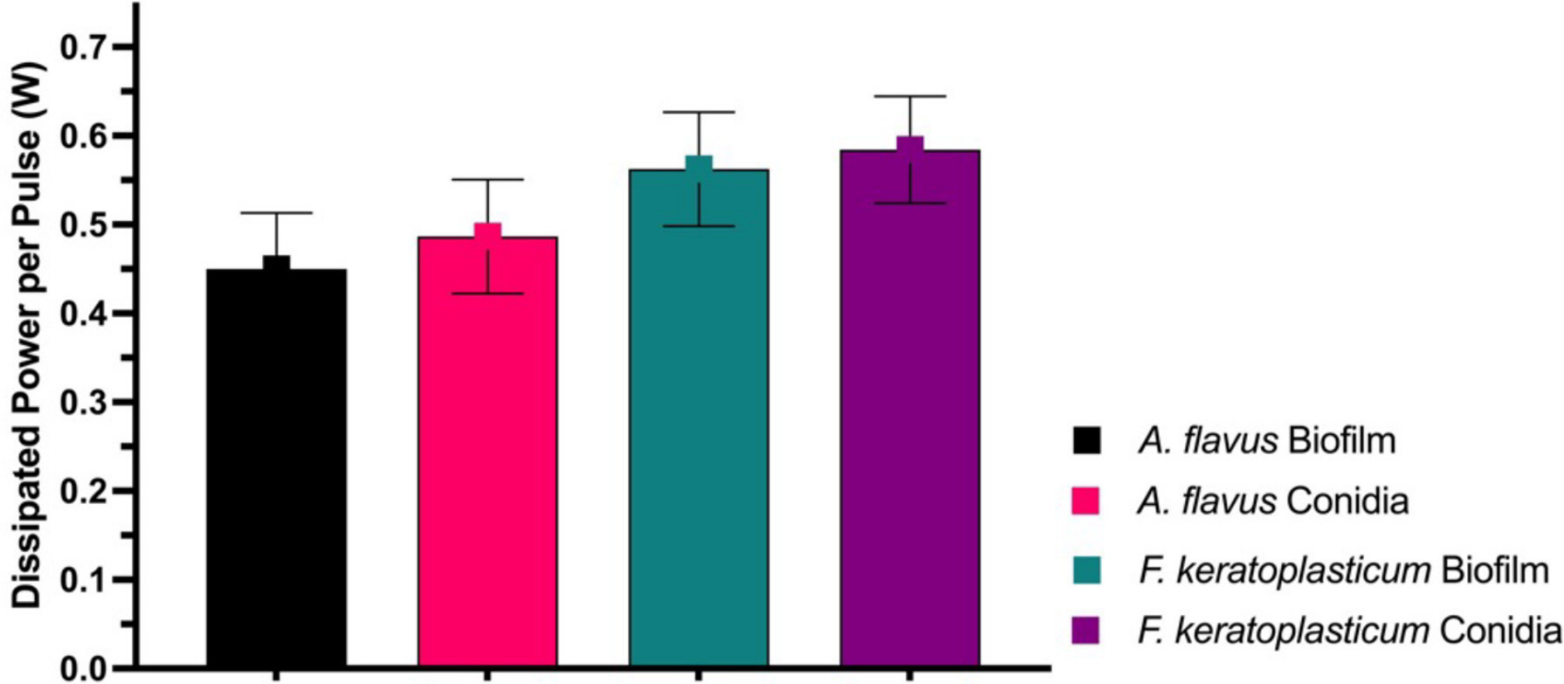
Volume DBD dissipated power per pulse (*W*) variation among *A. flavus* and *F. keratoplasticum* biofilms and conidial suspensions. Power measurements obtained during CAP treatment of *A. flavus* and *F. keratoplasticum* biofilms and conidial suspensions at an applied voltage of 22 kV_pp_, pulse repetition frequency of 300 Hz and discharge current of 20 mA (mean±sem; *n*=3).

The average dissipated power per pulse at an applied voltage of 22 kV_pp_ was calculated for CAP treatment of *A. flavus* and *F. keratoplasticum* biofilms and conidia (Fig. 9). Dissipated power was subjectively higher during the treatment of *F. keratoplasticum* than *A. flavus*. Additionally, dissipated power was subjectively higher during the treatment of conidia in aqueous suspension versus *in vitro* biofilms.

## Discussion

Using a microsecond-pulsed DBD device operating at 22 kV_pp_, 300 Hz and 20 mA, we achieved over 80 % reductions in metabolic activity and c.f.u. ml^−1^ of *A. flavus* biofilms *in vitro* with a 5-min CAP treatment. In comparison, Los *et al*. [27] reported an 88.3 % reduction in metabolic activity and a 2.3 log c.f.u. ml^−1^ decrease after 20 min of exposure to a high-voltage DBD operating at 80 kV_pp_ and 50 Hz, suggesting delayed inactivation at higher voltage. Conversely, Simoncicova *et al*. [29] exposed a monolayer of *A. flavus* mycelium to a diffuse coplanar DBD operating at 400 W and reported a 90 % reduction in dry mycelial weight after 15 s of CAP exposure. These varying results underscore a major challenge in interpreting CAP literature, as outcomes are heavily influenced by device design, power supply parameters, treatment duration and cultivation method. However, the specific influence of each of these factors on the antifungal effect of CAP remains unclear.

Literature assessing the correlation between operating power and fungal inactivation lacks comprehensive insights. Our study revealed improved fungal inactivation at lower (22 kV_pp_) than at higher power (27 kV_pp_). Higher-power treatment (27 kV_pp_) led to rapid initial reductions in c.f.u. ml^−1^ of *A. flavus* biofilms followed by a reduction in inactivation rate with prolonged exposure, while lower-power treatment (22 kV_pp_) exhibited an initial delay in effect but achieved greater maximal c.f.u. ml^−1^ reduction over time. This same trend was identified by Hojnik *et al*. [28] in treating *A. flavus* conidia at 5, 10 or 15 kV_pp_, with significant reductions in c.f.u. ml^−1^ at all powers after treatment for 240 s. In a display of rapid inactivation at high voltage, Wang *et al*. [38] reported complete inhibition of mycelial growth in *A. flavus* conidia after 30 s treatment at 70 kV_pp_. These results suggest that the fungal inactivation rate increases with voltage. However, when Ott *et al*. [39] similarly investigated CAP treatment of *A. flavus* conidia at 70 kV_pp_, 10 min of CAP treatment was required to achieve 95 % killing based on a SYTO9 Live/Dead assay. Future use of optical emission and absorption spectroscopy data are warranted to identify how input parameter alters relative RONS production to better elucidate the causality between varying input parameters and biological outcomes.

CAP treatment was also evaluated against *F. keratoplasticum* biofilms. Operating parameters of 22 kV_pp_, 300 Hz and 20 mA were selected due to the highest levels of inactivation among all parameters tested against *A. flavus* biofilms and resulted in the rapid inactivation of *F. keratoplasticum* biofilm. It is important to recognize that the ideal operating parameters for the inactivation of *F. keratoplasticum* may vary from those identified in *A. flavus*, and future research investigating the response of *F. keratoplasticum* to a spectrum of CAP power parameters will be critical in determining how to best optimize this technique for inactivation of *Fusarium* species. Despite this limitation, our results demonstrate that a single CAP operating parameter may be effectively applied to cause significant reductions in cell viability in two of the most prominent causative organisms of FK, each representing distinct fungal species belonging to phylogenetically different genera, classes, orders and families. A significant shortcoming of many antifungal drugs currently employed in the treatment of mixed *Aspergillus* and *Fusarium* infections is a relative lack of activity against the *Fusarium* [15,16, 18]. Importantly, we found CAP induced significantly greater reductions in fungal viability of *F. keratoplasticum* compared to *A. flavus*.

For the first time, we report greater dissipated power during CAP treatment of *F. keratoplasticum* compared to *A. flavus*, as well as when treating fungal conidia in aqueous suspension versus *in vitro* biofilms (Fig. 9). While improved CAP activity against *A. flavus* conidia compared to *A. flavus* biofilms has been previously observed by Los *et al*. [27], variation in dissipated power with target species has not been described. The cause of this difference in dissipated power remains unknown, but based on the mechanism(s) of action of CAP, it is plausible that differences in the composition and structure of the fungal cell wall and membrane play a significant role. The cell wall and membrane are well-established targets of CAP-generated RONS, including •OH, ^1^O_2_ and O_3_. These oxidants interact with the fungal cell membrane, inducing lipoperoxidation and increasing membrane permeability, which ultimately leads to cytosolic leakage and cell death [27,29,38]. Given the diverse composition of cell walls and membranes among fungal species, further investigation into the molecular and cellular interactions involved in CAP’s effects on different fungal species is warranted. Understanding how variations in the structural components of fungi contribute to their resistance or susceptibility to CAP treatment could provide valuable insights for optimizing CAP-based antifungal treatments.

Interestingly, we observed that CAP treatment of short duration (60 or 120 s) frequently resulted in enhanced metabolic activity compared to untreated controls. Improved susceptibility to antimicrobials is a well-documented result of enhancing microbial metabolic activity, especially among biofilm-encased microbes [40,41]. Indeed, enhancing microbial metabolism is a common mechanism of antimicrobial potentiation alongside interfering with microbial membrane permeabilization [41]. CAP-induced fungal membrane permeabilization is a well-documented effect in *A. flavus* [27,29, 38]. Coupled with enhanced microbial metabolic activity, as observed in this study and by Los *et al*. [27] in *A. flavus* biofilms, these observations reveal a potential use for low-dose CAP to potentiate antifungal drug treatment. While the impact of enhanced metabolic activity on antibiotic susceptibility is well-documented in bacteria, effects on fungal pathogens are less explored [40,41]. A thorough investigation into the antifungal drug susceptibility of CAP-treated fungi is essential to validate this potential application. Ultimately, future research identifying the mechanism(s) of CAP influence on fungal metabolism will be needed to fully realize this potential application.

In conclusion, the use of CAP as an antifungal treatment represents a novel, rapidly developing field of research. Though *A. flavus* has been extensively studied as a model system due to its significance as a world health threat to animals, humans and plants, variations in fungal cultivation and CAP delivery methods across existing literature hinder definitive conclusions on the antifungal effect of CAP on this species. Therefore, we chose to use *A. flavus* as a representative species for optimizing the device used in this study, with the objective of contributing to a comprehensive dataset that enables the identification of emerging trends in *A. flavus* inactivation. It is essential to recognize that further investigation into the response of *F. keratoplasticum* to varying CAP parameters, similar to those conducted in this and previous studies with *A. flavus*, will be crucial for advancing our understanding of optimizing CAP for species-specific treatment. At the same time, practical application of CAP as a medical antifungal intervention requires assessing whether a single dose can effectively target ecologically and phylogenetically diverse fungal species and morphologies, as we aimed to achieve in this study. Ultimately, the methodology employed here and the results obtained thereof contribute significantly to the validation of CAP as a potential broad-spectrum treatment. Based on the results of this study, CAP treatment may be able to address the need for a broad-spectrum antifungal intervention with efficacy against mature *Aspergillus* and *Fusarium* organisms. In particular, CAP treatment has the potential to dramatically improve treatment outcomes in fungal infections caused by *F. keratoplasticum* and potentially other species of *Fusarium*. In pursuit of further validation of a broad-spectrum application of CAP, future research will involve investigating the antifungal effect of a single power dose of CAP across a comprehensive and diverse catalogue of fungal species and strains.

## Conclusions

CAP exhibited potent antifungal effects against *A. flavus* and *F. keratoplasticum* conidia and biofilms *in vitro*. The antifungal effect of CAP was strongly time-dependent, influenced to a lesser degree by the operating power and morphology of the target organism.

## supplementary material

10.1099/jmm.0.001858 Fig. S1.

## Abbreviations

CAP: cold atmospheric plasma
CV: crystal violet
DBD: dielectric barrier discharge
FK: fungal keratitis
MLST: multi-locus sequence typing
RONS: reactive oxygen and nitrogen species
XTT: 2,3-bis-(2-metBis-(2-Methoxy-4-nitro-5-sulfophenyl)-2H-tetrazolium-5-carboxanilide, disodium salt
