# Supplementary material for: Cold atmospheric plasma inactivates Aspergillus flavus and Fusarium keratoplasticum biofilms and conidia in vitro
Source: J Med Microbiol. 2024 Jul 10;73(7):001858. doi: 10.1099/jmm.0.001858 (PMC11316566; doi:10.1099/jmm.0.001858)
Supplement: Fig. S1. [file jmm-73-01858-s001.pdf]

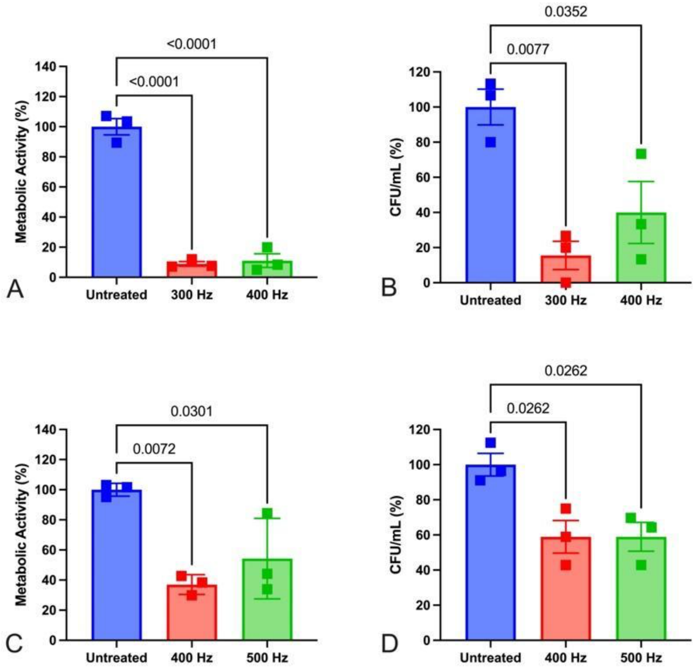

Supplemental Figure 1. Changing repetition frequency does not significantly alter CAP induced inactivation of *Aspergillus flavus* biofilms. (A) Metabolic activity and (B) CFU/mL following 300 s CAP treatment at 27 kV_{pp}, 300 or 400 Hz, and 60 mA. (C) Metabolic activity and (D) CFU/mL following 180 s CAP treatment at 26 kV_{pp}, 400 or 500 Hz, and 40 mA. Metabolic activity determined by XTT reduction, absorbance values normalized to untreated controls. CFU/mL normalized to untreated controls. Significant differences between all sample groups shown as p-value where indicated (mean ± SEM; n=3).
